# Job Satisfaction and Associated Factors among Scrub Nurses: Beyond the Surface

**DOI:** 10.3390/ijerph20247159

**Published:** 2023-12-08

**Authors:** Biljana Kurtović, Ilijana Bilješko Štrus

**Affiliations:** 1 Department of Nursing, University of Applied Health Sciences, 10000 Zagreb, Croatia; 2 Department of Nursing, Faculty of Health Studies, University of Rijeka, 51000 Rijeka, Croatia; 3 Instrumentation and Central Sterilization Unit, General Hospital Šibenik, 22000 Šibenik, Croatia; ilijana.biljesko@gmail.com

**Keywords:** perioperative nursing, job satisfaction, working conditions

## Abstract

Background: Job satisfaction is essential, especially among healthcare professionals like scrub nurses, who often face unique professional challenges. This study aimed to evaluate job satisfaction and its related factors among scrub nurses, shedding light on areas of improvement and highlighting the positive aspects of their roles. Methods: A detailed study was conducted among a cohort of 31 scrub nurses using a 28-question survey. The questionnaire gauged various aspects of job satisfaction, from work conditions to professional growth opportunities. Results: Notably, the participants showed the least satisfaction with hazard allowances (1.8 ± 1.1) and break durations (1.9 ± 1.3). However, high satisfaction was observed regarding resource availability (3.9 ± 1.0) and the quality of protective equipment (3.7 ± 1.0). The data also revealed that those with 6–10 years of experience were the most satisfied (3.5), while those with 16–20 years were less content (2.7). Conclusion: The study indicates the need for improvements in hazard allowances and break periods. Experience significantly influenced satisfaction, with mid-career nurses showing the highest contentment. Such insights can guide future policy and practice adjustments in healthcare settings.

## 1. Introduction

Job satisfaction can be broadly described as the attitudes, feelings, and beliefs individuals hold about their work. This construct of job satisfaction is multi-dimensional, encompassing both global dimensions (overall job contentment) and specific facets (satisfaction with aspects such as pay, progression, and peer relationships) [1]. Job dissatisfaction significantly impacts an individual’s mental and physical health, leading to chronic stress and reduced workplace efficiency. Addressing its root causes is crucial for maintaining individual well-being and enhancing overall workplace productivity and harmony [2]. Recent research has elucidated that work motivation has a negative and significant impact on job satisfaction, highlighting a critical aspect of employee engagement and organizational dynamics in various professional settings [3]. Extensive research shows that hospital nurses’ job satisfaction is influenced by a complex array of factors, including work environment, empowerment, commitments, stress, patient dynamics, and cultural background [4]. Understanding the factors influencing nurses’ job satisfaction is key to enhancing healthcare quality and productivity, necessitating that policymakers and employers improve work conditions for better employee well-being [5]. In recent years, the role of scrub nurses has become increasingly complex and demanding due to technological advancements in surgical procedures and evolving healthcare policies. Global trends in nursing job satisfaction reflect these changes, with various factors, such as workload, work–life balance, and professional development opportunities playing a crucial role. Furthermore, patient expectations in terms of care quality have also evolved, adding to the challenges faced by scrub nurses in maintaining job satisfaction. Understanding these global trends is essential for developing effective strategies to enhance job satisfaction among scrub nurses.

The importance of job satisfaction in the professional landscape has garnered significant attention in recent years. The European Working Conditions Survey (EWCS) by Eurofound reveals a definitive correlation between job satisfaction and employee financial compensation [6]. Compensation strategies significantly affect individual and collective employee contentment, especially in healthcare, where they deeply influence overall job satisfaction. Recent studies have demonstrated a positive correlation between patient satisfaction and aspects of nursing practice, such as job satisfaction and self-efficacy, while also showing a negative correlation with nurses’ turnover intention, thereby emphasizing the link between patient outcomes and nursing staff well-being [7]. These insights highlight the importance of effective human resource management in healthcare, where both the work environment and personal initiative are key in shaping professional outcomes [8]. Furthermore, another study indicates that nurses’ job satisfaction is shaped by multiple organizational factors, including empowerment, compensation, and work environment, pointing to the complexity influencing their motivation [9]. Additionally, demographic variables, like age, gender, marital status, educational attainment, and organizational position, further mold job satisfaction.

In analyzing job satisfaction trends among scrub nurses, it is crucial to consider the diverse cultural and economic contexts across countries. Developed nations with robust healthcare systems often report higher nurse satisfaction, attributed to better working conditions, training, and career opportunities [10]. In contrast, nurses in resource-constrained environments typically face challenges like overwhelming workloads, impacting satisfaction levels negatively [11]. Cultural values, particularly regarding the nursing profession and work–life balance, also play a significant role in shaping job satisfaction [12]. Furthermore, economic aspects, such as compensation and job security are key contributors, with better-funded healthcare systems tending to offer more favorable conditions for nurses. These global comparisons underscore the multifaceted nature of factors influencing job satisfaction, necessitating tailored strategies in different cultural and economic settings. 

As healthcare systems worldwide continue to evolve, the role of scrub nurses becomes increasingly pivotal. The interplay between their job satisfaction and the quality of patient care they provide cannot be overstated. Therefore, understanding the factors that influence their job satisfaction is crucial. This not only helps in improving their work conditions but also ensures the delivery of high-quality patient care. The need for comprehensive research in this area is more pressing than ever, as healthcare systems grapple with challenges like staff shortages and increasing patient loads. Maintaining nurses’ job satisfaction is vital in healthcare, as it affects productivity, absenteeism, and stress, directly impacting healthcare standards [8,13]. Systematic review findings emphasize the need for healthcare organizations to implement strategies improving nurses’ job satisfaction and reducing turnover, focusing on strategies that enhance intrinsic motivation [14]. Enhancing job satisfaction is crucial for better working conditions and retention in intensive care units, with a positive work environment reducing turnover intentions [15,16]. The global nursing shortage adds urgency to addressing nurses’ satisfaction with their work environment [16]. Understanding what drives nurses’ job satisfaction is essential for healthcare institutions’ smooth operation and high care standards, requiring employment strategies that attract and retain qualified staff [17]. Studies also show a link between job satisfaction and work efficiency, suggesting that dissatisfaction can diminish the supply of nurses in healthcare organizations [18].

In the operation room, scrub nurses are essential, skillfully blending technical and non-medical knowledge with psychomotor, procedural, relational, and communication skills for the successful orchestration of surgeries [19]. Scrub nurses’ effective communication, relational skills, adaptability, and commitment to continuous learning and evidence-based practice are essential for smooth surgical procedures, exemplary patient care, and ensuring patient safety in a rapidly evolving medical landscape [20]. The critical role of scrub nurses in the OR is highlighted by the severe consequences that can arise from even minor lapses in their duties, where a single oversight or miscommunication can lead to significant complications. Consequently, their mental and emotional well-being is paramount, not only for efficient surgical processes but also for their holistic health, as a satisfied and well-supported scrub nurse directly impacts the quality of patient care. This research seeks to deepen the understanding of the factors influencing scrub nurses’ job satisfaction, thereby furnishing insights to healthcare institutions on fostering a conducive environment for these nurses. The job satisfaction of scrub nurses is not just a matter of individual contentment but has far-reaching implications for the efficiency and effectiveness of healthcare systems. High job satisfaction can lead to better patient outcomes, lower turnover rates, and improved overall healthcare delivery. Thus, understanding and improving job satisfaction among scrub nurses is vital for the sustainability and advancement of healthcare services.

## 2. Materials and Methods

### 2.1. Study Design 

This study utilized a cross-sectional survey method to evaluate the job satisfaction of scrub nurses in operating rooms in June 2023.

### 2.2. Participants

The participants comprised 31 scrub nurses from the General Hospital Šibenik, Croatia. In this study on job satisfaction determinants in surgical suites, 81% of the participants were females and 19% were males, spanning an age range from 26 to over 46 years. With respect to marital status, a significant majority (97%) were married or in recognized unions, contrasting with 3% who were single. In the context of educational attainment, 61% had completed secondary education, 35% held advanced vocational qualifications, and a minority of 3% were tertiary degree holders. Regarding professional tenure, it was indicated that 52% had more than 20 years in the field. As for shift preferences, 45% were aligned with morning shifts or rotational 12 or 24 h schedules, while 10% were predominantly engaged in on-call and standby functions (Table 1).

### 2.3. Data Collection Instrument

The research tool was a modified questionnaire derived from the article *Job Satisfaction and Associated Factors among Nurses Working In the Operation Theater at Government Hospitals of Eastern Ethiopia, 2017* [21]. Permission was secured from the original authors for modification and use. The questionnaire was bifurcated into two sections: sociodemographic data and job satisfaction. The latter consisted of 20 questions from the initial questionnaire and an additional 8 custom questions. Respondents rated each item using a 6-point Likert scale, indicating varying levels of satisfaction: 1—very dissatisfied, 2—dissatisfied, 3—somewhat dissatisfied, 4—somewhat satisfied, 5—satisfied, and 6—very satisfied. The survey design was chosen for its comprehensive coverage of various facets of job satisfaction relevant to scrub nurses. It was adapted to better suit the specific context of our study setting, incorporating elements that reflect the unique challenges and opportunities faced by scrub nurses in our region. These adaptations were necessary to ensure that the survey accurately captured the nuances of the scrub nurses’ work environment and their perceptions of job satisfaction.

### 2.4. Data Analysis

Data processing was facilitated using the STATISTICA 11.0 software package (StatSoft Inc., Tulsa, OK, USA) In our analysis, we made a clear distinction between categorical variables and those measured on a Likert scale. Frequencies of categorical variables were presented as numbers and percentages to depict the distribution of responses. For variables measured on the Likert scale related to job satisfaction, we treated the responses as interval data. This approach enabled the calculation of the mean and standard deviation values of these items, providing a measure of central tendency and variability. This treatment of Likert scale data as interval data aligns with established practices in the field, particularly when the scale is presumed to have equidistant points between response options. Furthermore, to examine the relationship between predictive variables and overall job satisfaction, we employed the Pearson correlation test. This method was chosen due to its appropriateness for assessing linear relationships among interval data, ensuring a robust and relevant analysis of the correlations present in our study. Furthermore, we computed the mean and standard deviation of average job satisfaction in relation to sociodemographic variables, with results presented in tables and graphics for visual clarity.

Differences in responses between the two participant groups were analyzed using the Mann–Whitney U test, while the Kruskal–Wallis ANOVA test was applied for comparing responses across three or more groups. The impact of predictor variables on overall job satisfaction was examined using multiple regression analysis within a general regression model framework. The outcomes of these analytical methods were summarized and visualized using a Pareto diagram of t-values, with a *p*-value of less than 0.05 deemed statistically significant.

### 2.5. Ethical Considerations

The Ethics Committee of the hospital approved the study (approval number 01-14954/1-23). Before the commencement of the research, all participants were comprehensively briefed about the study’s objectives, ensuring they had a clear understanding of its purpose and what would be expected of them. Each participant was then provided with an informed consent form, detailing the procedures, potential risks, and benefits associated with the study. This step was crucial in establishing transparency and trust. By signing the informed consent, the participants acknowledged their willingness to take part in the research, asserting that their participation was entirely voluntary. Furthermore, they were assured of their right to withdraw from the study at any point without any repercussions. 

## 3. Results

Job satisfaction, gauged on a scale from 1 to 5, was scrutinized through 28 specific items/statements. The mean scores and standard deviations for each item/statement across categories are elucidated in Table 2, Table 3 and Table 4.

Regarding job satisfaction and associated factors, the mean scores oscillated between 1.8 and 3.9 out of a potential 5, contingent on the specific item/statement. The participants exhibited the lowest satisfaction scores concerning hazard pay for scrub nurses in the operating theatre (1.8 ± 1.1) and the duration of breaks (1.9 ± 1.3). Conversely, the highest satisfaction was attributed to the availability of resources and supplies (3.9 ± 1.0) and both the quantity and quality of protective gear (3.7 ± 1.0) (Table 2). The varying levels of job satisfaction across different factors reflect the complex nature of the nursing role in contemporary healthcare settings. While certain aspects, such as resource availability, are satisfactory, areas like hazard pay and break durations reveal critical gaps. These disparities highlight the multifaceted challenges faced by scrub nurses and underscore the importance of a holistic approach to enhancing their work environment.

**Table 2:** Job-related satisfaction metrics.

Observed Variable	$\bar{X}$	SD
1. Availability of resources and supplies	3.9	1.0
2. Opportunity for advancement	2.8	1.3
3. Opportunity for professional development or education	2.9	1.4
4. Salary	2.5	1.2
5. Workload	3.0	1.6
6. Providing quality care to patients	3.6	1.1
7. Patient treatment outcomes	3.8	1.0
8. Physical conditions of the workplace	3.2	1.3
9. Freedom to choose one’s own way of working	2.8	1.3
10. Recognition received for good work	2.4	1.4
11. Financial compensation for work	2.0	1.3
12. Hazard allowance for scrub nurse	1.8	1.1
13. Break	1.9	1.3
14. Quantity and quality of protective equipment	3.7	1.0
15. Method of dispatching non-sterile material and delivering sterile material to the operating room	2.8	1.4

In the context of satisfaction related to relationships, scrub nurses expressed the least satisfaction with the perspective on the centralization of instrument technicians in a singular operational unit (2.0 ± 1.2). However, they manifested high satisfaction with aspects of assisting others (3.9 ± 1.1) and patient treatment outcomes (3.8 ± 1.0) (Table 3).

**Table 3:** Satisfaction concerning interpersonal relations.

Observed Variable	$\bar{X}$	SD
1. Assisting others	3.9	1.1
2. Attitudes of doctors towards scrub nurses in the operating room	3.5	1.2
3. Public awareness of the role of scrub nurses in operating rooms	2.3	1.3
4. Amount of responsibility of scrub nurses in the operating room	3.4	1.4
5. Support scrub nurses receive in the operating room	2.8	1.1
6. Your healthcare worker status	3.0	1.3
7. Your feelings about the job itself	3.3	1.4
8. Motivation to work	3.1	1.4
9. Collaboration with other scrub nurses in different operating rooms	3.6	1.0
10. Opinion of scrub nurses about centralization of scrub nurses in a single unit.	2.0	1.2

When evaluating satisfaction regarding workplace communication, scrub nurses and instrument technicians generally expressed contentment (Table 4).

**Table 4:** Satisfaction pertaining to communication.

Observed Variable	$\bar{X}$	SD
1. Communication among scrub nurses in different operating rooms	3.3	1.1
2. Communication between scrub nurse and physicians	3.6	1.1
3. Communication of scrub nurses within individual operating rooms.	3.5	1.0

Considering the average scores of all 28 questions/statements, the overall job satisfaction was rated at 3.0 ± 0.7 out of a possible 5. Differences in the ratings of average job satisfaction and associated factors based on the socio-demographic characteristics of the population were marginal. There was no discernible difference in satisfaction between individuals with secondary and higher vocational education, though there was slightly higher satisfaction among those with tertiary education. Satisfaction levels for those working morning shifts and those on duty and standby were similarly assessed, with a marginally lower average rating compared to participants working in rotating shifts. The most noticeable variations in ratings were evident when considering years of work experience, with the most satisfied participants having a work tenure of 6 to 10 years (3.5), and the least satisfied having a tenure of 16 to 20 years (2.7) (Table 5).

There is virtually no difference in the scores between men and women. Respondents under the age of 45 years exhibited slightly higher satisfaction (3.1) compared to those aged over 45 years (2.9). Additionally, individuals in marital relationships demonstrated marginally higher satisfaction than those who were single. Neither the Mann–Whitney U test nor the Kruskal–Wallis ANOVA test revealed a statistically significant difference between the groups for any socio-demographic variable. The results of the multiple regression analysis exploring the influence of predictor variables on average job satisfaction are presented in Table 6. It is evident that there was no correlation (r = 0.10; *p* = 0.9997) between the predictor variables and average job satisfaction, as indicated by the beta coefficients and their significance.

## 4. Discussion

In a study examining job satisfaction and its associated factors among scrub nurses at the General Hospital Šibenik, a total of 31 participants were involved, comprising 81% women and 19% men. The evaluation of job satisfaction spanned a scale from 1 to 5, operationalized through 28 distinct questions/statements. The mean job satisfaction score was 3.0 ± 0.7, indicating a moderate level of satisfaction among the participants. The results indicate that the socio-demographic attributes of the participants did not exert a significant influence on the job satisfaction of scrub nurses. This finding suggests that factors beyond demographic characteristics, such as workplace environment and job roles, may play a more pivotal role in determining job satisfaction.

Our study contributes to the broader understanding of job satisfaction in nursing by highlighting specific factors within a specialized hospital department. The study’s outcomes underscore the critical factors that influence job satisfaction, which has implications for nursing management and policy-making. By focusing on these factors, healthcare administrators can develop more effective strategies for improving the work environment and enhancing job satisfaction. Furthermore, our research highlights the need for the continuous assessment of job satisfaction within healthcare settings. This is particularly relevant in the current climate, where the retention of nursing staff is a pressing concern globally. 

Our study reveals that scrub nurses’ main areas of discontent are related to hazard pay and insufficient break times, aligning with the broader literature on nurse job satisfaction and remuneration but with unique aspects specific to their operating room environment. This is echoed in the findings of Eskola et al., who noted general dissatisfaction in healthcare professionals regarding remuneration, a sentiment that resonates in our study and can significantly affect job satisfaction, morale, and retention in high-stress environments like the operating room [22]. Further supporting this trend, a study by Zhou and Gong [23] from China found that low salaries were the second biggest reason for job stress in the operating room. While this study was conducted in a different geographical and cultural context, it underscores the universality of the challenge faced by healthcare professionals, especially those working in critical areas like the OR. 

Our analysis also revealed demographic nuances in job satisfaction among scrub nurses. While overall satisfaction levels were moderate, we noted that younger nurses and those in the early to mid-stages of their careers tended to report higher satisfaction. This trend suggests that factors such as career development opportunities, adaptability to changing work environments, and evolving professional roles might differentially impact nurses based on their career stage and age. The observed demographic variations in job satisfaction underscore the need for healthcare policies that are sensitive to the diverse needs of scrub nurses. For instance, younger nurses and those in the early stages of their careers may benefit more from opportunities for skill development and mentorship. In contrast, more experienced nurses might value initiatives that recognize their expertise and contributions, such as advanced professional roles or leadership opportunities.

The impact of workday breaks on job satisfaction is particularly noteworthy. Labor laws mandate workday breaks to enhance employee health and productivity, but scrub nurses often miss these due to increased surgeries and overtime [24]. This highlights a critical area for policy intervention, particularly in revising labor laws related to scrub nurse remuneration and work conditions. An Eastern Ethiopian study highlighted job satisfaction’s crucial role in nurses’ professional lives, affecting patient safety, productivity, performance, care quality, retention, and loyalty [21]. This research, focusing on scrub nurses, found a divided sentiment, with 48% satisfied (8.2% completely, 38.8% moderately) and 52% dissatisfied, identifying the hospital’s nature as a key factor in job satisfaction [21]. An Iranian study underscores the importance of identifying job satisfaction determinants in healthcare, finding that job security, qualifications, and pay are key drivers, yet only a minority of nurses reported moderate to high satisfaction [25]. Factors like employment type, tenure, and work hours, with significant *p*-values, emerged as influential, highlighting the need to understand and address these aspects for a motivated workforce [25]. This research emphasizes the complexity of job satisfaction and its impact on healthcare efficacy and personal career trajectories [25]. A study involving 60 perioperative nurses in a tertiary hospital found diverse levels of job satisfaction, with working hours and job security as key predictors [26]. This contrasts with our findings, where dissatisfaction centered around hazard pay and break duration, but satisfaction was high regarding resource availability and quality of protective equipment.

This synthesis aims to consolidate findings from a range of studies, providing a comprehensive view of job satisfaction among scrub nurses in various settings. The heterogeneity in findings can be ascribed to variations in institutional governance, regional policies, cultural distinctions, and organizational dynamics. These insights offer a roadmap for potential interventions aimed at enhancing job satisfaction among scrub nurses, which can positively influence patient care outcomes.

Our findings point to the need for ongoing monitoring and evaluation of job satisfaction among scrub nurses, especially as the healthcare sector continues to evolve. Regular assessment and proactive measures are essential in addressing issues as they arise, ensuring scrub nurses remain satisfied and motivated in their roles. 

This study has several limitations. Firstly, while the sample size of 31 participants may seem limited, it is reflective of the scrub nursing population in the General Hospital Šibenik, thus providing a comprehensive view of this specific group. However, the use of self-reporting for measuring job satisfaction might introduce potential biases, such as socially desirable responses or recall inaccuracies. The study’s geographical confinement to one hospital may impact the external validity and hinder the generalizability of results to different settings or regions. It is important to acknowledge that the correlation findings in this study may be influenced by our sample size. While the sample size was deemed appropriate for the exploratory nature of our research, future studies with larger cohorts could provide more robust data, potentially revealing subtler effects not detected in this study. Exclusively employing quantitative data might overlook nuanced insights that could be gathered from qualitative methods like interviews. Additionally, the cross-sectional design provides only a snapshot of job satisfaction, not capturing potential longitudinal changes. Voluntary participation could also potentially introduce a sampling bias if certain groups were more inclined to participate.

## 5. Conclusions

Following the data analysis in this study conducted at the General Hospital of Šibenik, it was ascertained that respondents demonstrated marked dissatisfaction concerning the hazard pay for scrub nurses and the duration of breaks. Conversely, there was pronounced satisfaction associated with the availability of resources, collaborative efforts, and patient treatment outcomes. Highlighted areas of dissatisfaction accentuate the necessity for systematic evaluations of job satisfaction to inform interventions aimed at enhancing the quality-of-service delivery and employee contentment.

Future implications for clinical practice suggest revisiting policy guidelines, especially those related to training and compensation. Subsequent research endeavors should emphasize a multifaceted approach, considering varied healthcare settings and integrating qualitative methodologies to elucidate underlying factors of job satisfaction and their ramifications on healthcare outcomes. 

Our study provides valuable insights into the factors affecting job satisfaction among scrub nurses. These insights have significant implications for nursing management and healthcare policies, particularly in terms of developing strategies to enhance job satisfaction, which in turn, can positively affect patient care and healthcare outcomes. Our findings advocate for policy revisions that address specific dissatisfaction areas while reinforcing those contributing to high satisfaction levels.

## Figures and Tables

**Table 1:** Sociodemographic characteristics of participants (*n* = 31).

Variable	Group	*n*	%
Gender	Women	25	81
	Men	6	19
Age (years)	26–35	9	29
	36–45	9	29
	>46	13	42
Marital status	Married	30	97
	Single	1	3
Educational level	Secondary school education	19	61
	Bachelor’s degree	11	35
	Master’s degree	1	3
Work experience (years)	6–10	5	16
	11–15	7	23
	16–20	3	10
	>20	16	52
Working hours	Morning shift	14	45
	Shift work (12, 24)	14	45
	On-call and standby duty	3	10

**Table 5:** Average job satisfaction and associated factors among scrub nurses in the operating room based on socio-demographic characteristics of the population.

Variable	Group	$\bar{X}$	SD	*p*
Gender	Women	3.0	0.7	0.9594
	Men	3.0	0.7	
Age (years)	26–35	3.1	0.8	0.7923
	36–45	3.1	0.7	
	>46	2.9	0.7	
Marital status	Married	3.0	0.7	0.8693
	Single	2.9	0.0	
Educational level	Secondary school education	3.0	0.7	0.9836
	Bachelor’s degree	3.0	0.9	
	Master’s degree	2.9	0.0	
Work experience (years)	6–10	3.5	0.6	0.3631
	11–15	2.8	0.7	
	16–20	2.7	0.6	
	>20	3.0	0.7	
Working hours	Morning shift	3.0	0.8	0.9361
	Shift work (12, 24)	3.1	0.8	
	On-call and standby duty	3.0	0.3	

**Table 6:** Results of the multiple regression analysis examining the impact of predictor variables on average job satisfaction and associated factors among scrub nurses in the operating room.

Predictor Variable	ß	*p*
Age	0.03	0.8908
Gender	0.00	0.9888
Marital status	0.03	0.8721
Work experience	0.08	0.7117
Educational level	0.02	0.9438
Working hours	0.01	0.9791
	r = 0.10; *p* = 0.9997	

Note: r—correlation coefficient; *p*—significance level; ß—beta coefficient representing the individual contribution of each variable to the overall correlation.

## Data Availability

The data and the questionnaires of the study are available upon request from the corresponding author.
